# Recent achievements obtained by chloroplast transformation

**DOI:** 10.1186/s13007-017-0179-1

**Published:** 2017-04-19

**Authors:** Muhamed Adem, Dereje Beyene, Tileye Feyissa

**Affiliations:** 1 Department of Microbial, Cellular and Molecular Biology, College of Natural and Computational Sciences, Addis Ababa University, P.O. Box. 1176, Addis Ababa, Ethiopia; 2 Department of Forestry, School of Agriculture and Natural Resources, Madawalabu University, P.O. Box 247, Bale Robe, Oromiya Ethiopia; 3 Institute of Biotechnology, College of Natural and Computational Sciences, Addis Ababa University, Addis Ababa, Ethiopia

**Keywords:** Chloroplast transformation, Novel traits, Homologus recombination, Transgene, Regulatory sequences

## Abstract

Chloroplasts play a great role for sustained wellbeing of life on the planet. They have the power and raw materials that can be used as sophisticated biological factories. They are rich in energy as they have lots of pigment-protein complexes capable of collecting sunlight, in sugar produced by photosynthesis and in minerals imported from the plant cell. Chloroplast genome transformation offers multiple advantages over nuclear genome which among others, include: integration of the transgene via homologus recombination that enables to eliminate gene silencing and position effect, higher level of transgene expression resulting into higher accumulations of foreign proteins, and significant reduction in environmental dispersion of the transgene due to maternal inheritance which helps to minimize the major critic of plant genetic engineering. Chloroplast genetic engineering has made fruit full progresses in the development of plants resistance to various stresses, phytoremediation of toxic metals, and production of vaccine antigens, biopharmaceuticals, biofuels, biomaterials and industrial enzymes. Although successful results have been achieved, there are still difficulties impeding full potential exploitation and expansion of chloroplast transformation technology to economical plants. These include, lack of species specific regulatory sequences, problem of selection and shoot regeneration, and massive expression of foreign genes resulting in phenotypic alterations of transplastomic plants. The aim of this review is to critically recapitulate the latest development of chloroplast transformation with special focus on the different traits of economic interest.

## Background

World population is expected to rise to 9.2 billion in 2050. In order to feed the rising population food production has to grow in parallel. The problem is that arable land is exploited to its potential (High Level Expert Forum, FAO, October 2009; http://www.fao.org). Advancement in agricultural biotechnology particularly plant genetic engineering is believed to boost crop productivity. Due to enormous rewards crucial traits have been engineered via chloroplast genome instead of nuclear genome. It is amazing that more than 120 genes from various sources have been well integrated and expressed via the chloroplast genome for various applications. Aims of these applications include, developing crops with high levels of resistance to insects, bacterial, fungal and viral diseases, different types of herbicides, drought, salt and cold tolerance, cytoplasmic male sterility, metabolic engineering, phytoremediation of toxic metals and production of many vaccine antigens, biopharmaceuticals, industrial enzymes and biofuels [1–5].

Chloroplasts originated from endosymbiosis around 1.5 billion years ago, when a cyanobacterial cell was engulfed by heterotrophic eukaryote [6, 15]. Chloroplast organelle of plants and algal cells evolved from photosynthetic bacteria living inside the primitive ancestors of plant cells [7, 8]. Chloroplast gene products are not only homologus to the present-day cyanobacteria but the arrangement and expression of genes also reflect the prokaryotic ancestry of chloroplasts. They possess multiple copies of a small circular genome with 100–250 genes and their genome size varies between species, ranging from 107 kb (*Cathaya argyrophylla*) to 218 kb (*Pelargonium*) and maternally inherited in angiosperm plants [5]. There is a strong believe that the action of gene transfer and genome streamlining resulted into a drastic shrinkage of the genome of cyanobacterial endosymbiont where thousands of genes disappeared and were either transferred to nucleus or lost. Consequently, modern-day chloroplast genomes of photosynthetic eukaryotes are much reduced [9, 10].

The high ploidy number of the plastid genome and compartmentalization of proteins allow high levels of foreign protein expression from 5 to 40% total soluble protein [11] and up to 70% total soluble protein in Tobacco [2, 3, 12]. Moreover, nuclear encoded proteins are also accumulated at high level inside the chloroplast, although the ploidy level is not as high as chloroplast encoded proteins. That is why recent advancement in plant biotechnology has proved the use of chloroplasts as excellent ideal host for conferring agronomic traits and production of biopharmaceuticals, biomaterials and industrial enzymes [13]. Chloroplast genetic engineering has enormous advantages over nuclear transformation as well explained in Table 1 [1, 5, 14–25].

**Table 1:** Comparative advantages of chloroplast genome over nuclear genome

Chloroplast transformation	Nuclear transformation
Reduced of gene dispersal in the environment due to maternal inheritance	There is gene dispersal in the environment due to its parental nature
Multiple copy (high ploidy) of plastids results higher expression and accumulation of foreign proteins	Nuclear is not in high ploidy results lower expression and accumulation of foreign proteins
Efficient multiple gene expression in single transformation event	Efficiency of single transformation for multiple gene expression is very poor
Single promoter for expression of multi-subunit complex protein from polycistronic mRNAs	Several promoters for each genes to drive expression of respective subunits
Simultaneous expression of several genes as it contains prokaryotic gene expression system	Do not have prokaryotic expression system can’t undergo simultaneous expression of several genes
Homologous recombination avoids position effects and gene silencing	Random integration presents position effects and gene silencing

### Chloroplast transformation

**Fig. 1:**
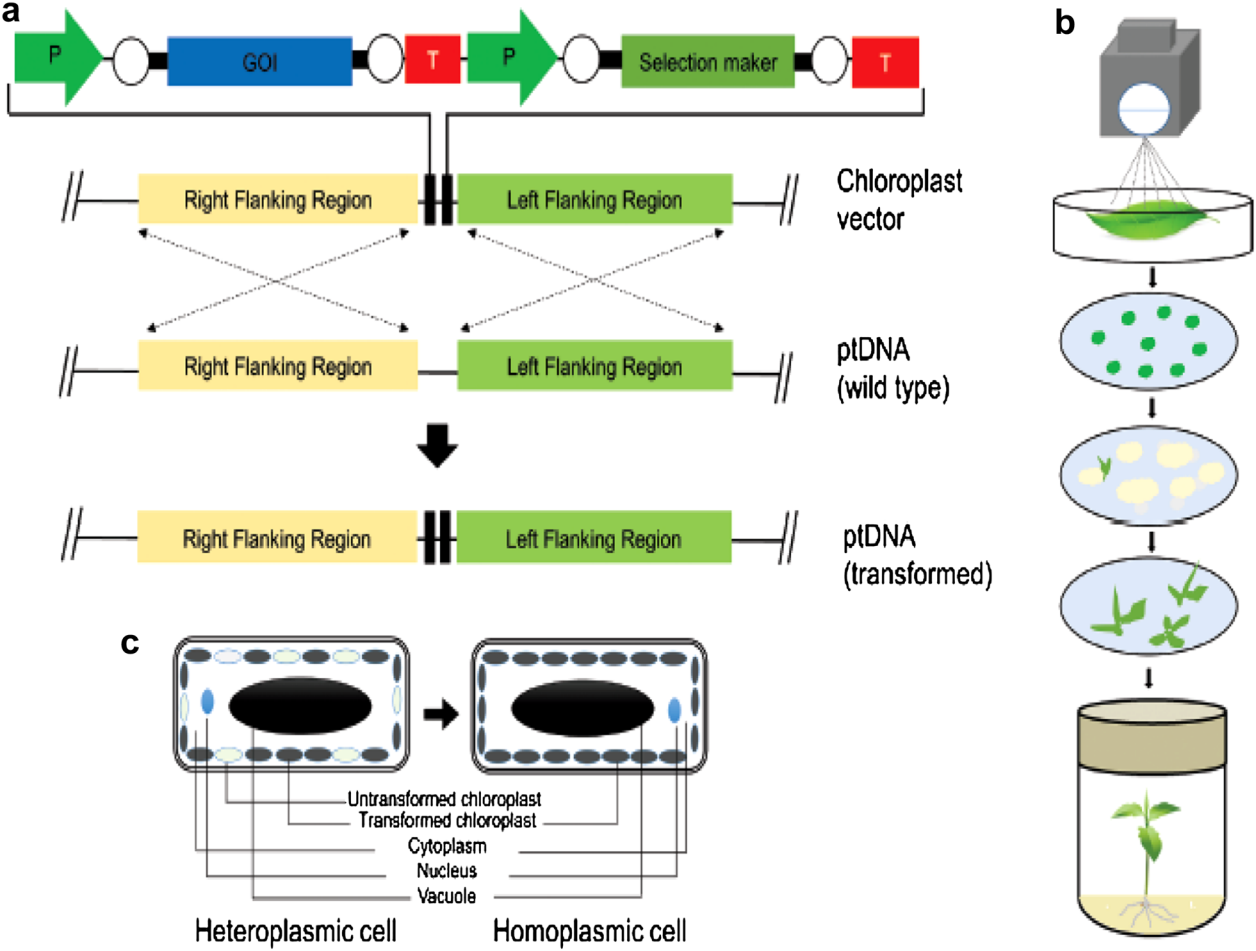
Diagrammatic representation of the processes for chloroplast genome transformation. **a** Basic design of a typical vector for transforming the plastid genome. Both the expression cassette and the selection cassette are placed between the two plastid regions. These flanking regions are taken from the wild-type plastid genome of a plant species whose plastome is to be manipulated, to allow a crossover event take place to integrate DNA sequences between them. *Green arrows* in the chloroplast expression vector represent promoters (P) and the direction of transcription, whereas terminators (T) are indicated by *red rectangles*. The untranslated regions are represented by *white circles*. The *thin dotted lines with arrows* indicate homologous recombination. **b** Delivery of transforming plasmids into chloroplasts in leaf cells using a particle delivery system. The plasmid DNA is coated on the surface of the microparticles of either gold or tungsten and then shot on to the abaxial surface of 4- to 6-week-old sterile leaves using a gene gun. The bombarded leaves are incubated for 48 h in the dark, cut into small discs and placed on regeneration medium supplemented with the appropriate antibiotic and hormones. Primary shoots generally arise within 2–3 months. **c** The process of recovering a stable homoplasmic transplastomic plant line. Initially, only a few copies of the plastome are transformed, and therefore the explant contains a mixture of both transformed as well as untransformed copies, a state known as heteroplasmy. The wild-type copies (indicated by *light-coloured ovals*) are sorted out gradually by repeating two or three regeneration cycles under selection to reach homoplasmy, a state where all copies of the plastome are transformed (indicated by *dark grey ovals*). Adopted from Ref. Ahmad et al. [113]

Multistep processes are involved to achieve chloroplast transformation. Species specific or heterologous chloroplast transformation vectors are developed in a manner that flanks the foreign genes and insert them through homologous recombination at predetermined and precise location in the plastome [26]. When the foreign DNA is delivered into plasmids, initially only a few copies of the plastome are transformed resulting in-to heteroplasmic state. Then, through sub-culturing the bombarded explants in vitro under selection all copies of the plastome contains the transgene leading to the state of homoplsamy, where all the plastomes of the chloroplasts present in the cell are transformed (Fig. 1). Generally, three key conditions have to be full-filled to achieve plastid transformation: (1) a robust method of DNA delivery into the chloroplast, (2) the presence of active homologous recombination machinery in the plastid, and (3) the availability of highly efficient selection and regeneration protocols for transplastomic cells [11, 27].

Transformation is highly efficient when there is complete homology of plastid DNA flanking sequences. For successful transformation, it is critical to identify promoters, 5′-UTRs, 3′-UTRs and insertion sites as indicated in Table 2. Complete chloroplast genome sequences are essential for integration of the transgene at optimal site via homologus recombination and to identify endogenous regulatory sequences for optimal transgene expression [28, 29].

**Table 2:** Commonly used promoters, un-translated regions and insertion sites for chloroplast transformation as avowed in [25, 117]

Promoter	5′-UTRs	3′-UTRs	Popular insertion sites
P*psbA*	*Ggagg*	*rbcL*	*rbcL*-*accD*
P*rna*	T_7_G_10_	*rps16*	*Trnl*-*trnA*
P*rbcL*	*rbcL*	*petD*	*rp132*-*trnL*
*psaA*	*atpB*	*psbA*	*petA*-*psbJ*
*atpI*	*psbA*		*3’rps12/7*-*trnV*
	*cry2a*		*Trn16/V*-*3’rps12/7*
			*23srrnA*-*16srrnA*
			*trnfM*-*trnG*
			*atpB*-*rbcL*
			*trN*-*trnR*
			*Ycf3*-*trnS*
			*rps7*-*ndhB*

Plastid transformation was first achieved in unicellular algae called *Chlamydomonas reindhartii* [30]. Tobacco was the first higher plant in which chloroplast transformation was successfully performed [31, 32]. Similarly, a protocol for plastid transformation of an elite rapeseed cultivar (*Brassica napus* L.) has been developed [33].

### Traits of interest for chloroplast transformation

#### Conferring agronomic traits

Researchers have successfully engineered different genes on chloroplasts to confer agronomic traits of interest. For instance simultaneous expression of protease inhibitors and chitinase have been employed to develop multiple biotic and abiotic stresses resistant plants, particularly tobacco [34]. Economical agronomic traits, such as herbicide resistance, insect resistance and tolerance to drought and salt, have already been engineered via the plastid genome [35]. The dominant trait that attracted the most attention for plastid transformation has been herbicide tolerance [11, 36–38]. The production of plants resistant to high level of glyphosate was achieved through biolistic transformation of plastids by introduction of a mutated herbicide-tolerant gene coding for EPSP synthase [11] (Table 3).

**Table 3:** Agronomic traits engineered via chloroplast genome

Site of integration	Regulatory sequences	Transgene/s	Efficiency of expression	Enhanced traits	References
rbcL/accD	Prrn/rbcL 3′	*panD*	>4-fold β-alanine	Tolerance to high-temperature stress	[39]
trnI/trnA	Prrn/ggagg/psbA	*tps1*	>169-fold transcript	Drought tolerance: growth in 6% polyethylene glycol and rehydration after 24 days of drought	[40]
rbcL/rbcL	psbA/psbA/3′rbL	*Hppd*	5% TSP	Resistance to herbicide	[41]
trnI/trnA	Prrn/T7 10/rps16	*Badh*	93–101 μM g^−1^ FW	Salt tolerance: carrot plants survived up to 400 mM NaCl	[42]
trnfM/trnG	atpI/rps16	*Lycopene β*-*cyclase*	0.28 mg g^−1^ DW	Herbicide resistance and triggers conversion of lycopene	[43]
rbcL/accD	Prrn/ggagg/psbA	*EPSPS/aroA*	NR	Resistance to glyphosate (>5 mM)	[32]
prs14/trnG	Prrn/T7 g10/TrbcL	*HTP*, *TCY*, *TMT*	NR	Cold-stress tolerance and increase in vitamin E in fruit	[44]
trnV/rps7/12	Prrn/Trps16	*EPSPS*	>10% TSP	Resistance to the herbicide glyphosate	[45]
trnV/rps12/7	Prrn/TrbcL	*b*-*bar1*	>7% TSP	Resistance to the herbicide phosphinothricin	[46]
trnI/trnA	Prrn/psbA/psbA	*phaA*	14.71 β-ketothiolase mg^−1^ FW	Engineered cytoplasmic male sterility	[47]
trnI/trnA	Prrn/T7 g 10/TpsbA	*γ*-*TMT*	>7.7% TSP	Increased salt tolerance and enhanced accumulationof ɑ-tocopherol in seeds	[48]
trnI/trnA	T7g10 or psbA	*RbcS*	>150-fold RbcS transcript	Restoration of RuBisCO activity in rbcS mutants	[49]
rbcL/accD	Prrn/ggagg/psbA	*cry2Aa2*	2–3% of TSP	Resistance to *Heliothis virescens*, *Helicoverpa zea*, and *Spodoptera exigua*	[50]
trnV/3′rps12	prrn T7G10/rps12	*Trx f*, *Trx m*	NR	Starch synthesis	[51]
trnI/trnA	5′psbA/3′psbA	*ubiC*	25% DW	250-fold higher pHBA polymer accumulation than nuclear transgenic lines	[52]
rbcL/accD	PpsbA/Trsp16	*TC*, *γ* -*TMT*	3 nmol h^−1^ mg^−1^ FW	Vitamin E accumulation in tobacco and lettuce	[53]
trnV/orf708	psbA/psbA/psbA	*BicA*	~0.1% TSP	CO_2_ capture within leaf chloroplasts	[54]
trnV/rps12/7	Prrn/rbcL/rps16	*cry1A(c)*	3–5% of TSP	Resistance to larvae of *Heliothis virescens*, *Helicoverpa zea*, and *Spodoptera exigua*	[55]
rbcL/accD	Prrn/Trps16	*CrtZ*, *CrtW*	NR	Accumulation of astaxanthin fatty acid esters in lettuce	[56]
trnV/rps12/7	Prrn/T7gene10/rbcL	*cry1Ab*	NR	Resistance to caterpillar of *Anticarsia gemmatalis*	[57]
trnI/trnA	Prrn/Trps16	*MSI*-*99*	89.75 μg g^−1^ FW	Resistance against rice blast fungus	[30]
trnI/trnA	*Prrn/TpsbA*	sporamin1, CeCPI2, and chitinase2	0.85–1% TSP	Resistance against *Spodoptera litura* and *Spodoptera exigua* leaf spot, as well as soft rot diseases	[58]
trnI/trnA	Prrn/ggagg/psbA	*Bt cry2Aa2 operon*	45.3% TSP	100% mortality of cotton bollworm, beet armyworm; cuboidal Bt crystals formation	[59]
trnI/trnA	Prrn/ggagg/psbA	*msi*-*99*	21–43% TSP	Resistance to in planta challenge of *Aspergillus flavus*, *Fusarium moniliforme*, *Verticillium dahlia*, and *Colletotrichum destructivum*	[60]
trnI/trnA	Prrn/ggagg/rbcL	*Bt cry9Aa2*	~10% of TSP	Resistance to *Phthorimaea operculella*	[61]
trnI/trnA	Prrn/psbA/psbA	*Cpo*	NR	Resistance to fungal pathogens in vitro (*Fusarium verticillioides*, and *Verticillium dahliae*) and in planta (*Alternaria alternata*)	[62]
trnI/trnA	5′psbA/3′psbA	*PelB1*, *PelD2*	~2.42 units mg^−1^ FW	Resistance against Erwinia soft rot	[63]
trnI/trnA	5′psbA/3′	*RC1011*, *PG12*	17–38% TSP	Resistance to Erwinia soft rot and tobacco mosaic virus	[64]
trnI/trnA	5′psbA/3′psbA	*Pta*	7.1–9.2% TSP	Broad-spectrum resistance against viral/bacterial/phloem-feeding insects	[51]
trnI/trnA	5′psbA/3′psbA	*Bgl*-*1*	>160-fold enzyme	Resistance against whitefly and aphid	[65]

### Production of vaccine antigens and biopharmaceuticals

It is believed that more than 90% of the global population cannot afford insulin, a drug needed to treat the global diabetes epidemic [5]. The high cost of protein drugs is due to their production in prohibitively expensive fermentation systems, prohibitively expensive purification from host proteins, the need for refrigerated storage and transport, and the short shelf-life of the final product [66, 67]. Protein drugs made by plant chloroplasts overcome most of these challenges as they do not require such expensive production process and can be stored without losing efficacy [68, 69] As listed in Table 4 numerous vaccine antigens and biopharmaceuticals have been engineered via chloroplast genome of higher plants.

**Table 4:** Vaccine antigens and biopharmaceuticals engineered via chloroplast genome of higher plants

Traits	Gene	Expression	Host plant	References
HIV/AIDS	*gp120*, *gp41*	16 μg g^−1^ FW	Tobacco	[70]
Human papiloma virus	*GUS*-*E7*	3–4% TSB	Tobacco	[71]
Polio virus	*CTB*-*VP1*	4–5% TSP	Tobacco	[72]
Tuberculosis antigens	*CTB*-*SAT6CTB*-*Mtb72F*	7.5% TSP	Tobacco	[73]
	*CTB*-*ESAT6*	0.75% TSP	Lettuce	[73]
Bacterial	*Pa*	2.5–4% TSP	Tobacco	[74]
Dengue virus	*EDIII*	0.8–1.6 TSP	Tobacco	[75]
Bacterial phage lytic `protein	*plyGBS*	>70% TSP	Tobacco	[76]
Pompe disease	*CTB*-*GAA*	0.1–0.2 TLP	Tobacco	[77]
Thioredoxin 1	*hTx1*	15 TSP	Lettuce	[78]
Insulin liken growth factors	*IGF*-*1n*	32% TSP	Tobacco	[79]
Endolysin Cpl-1	*Cpl*-*1*	10% TSP	Tobacco	[80]
Interferon-α2b(IFN-α2b)	*IFN*-*a2b*	21% TSP	Tobacco	[81]
Basic fibroblast growth factor (bFGF)	*bFGF*	0.1% TSP	Tobacco	[82]

Among plant plastids, tobacco plastid has been engineered to express the *E7 HPV* type 16 protein, which is an attractive candidate for anticancer vaccine development [83]. The main factor why plant plastids are chosen as better bioreactors is due to the ability of plants to correctly carry out post-translation modifications such as phosphorylation, amidation, proper folding, formation of disulfide bonds and the assembly of complex multi-subunit proteins. Microorganisms are also used for large-scale industrial applications of recombinant protein production, but cannot carryout post-translational modifications [35]. The hyper-expression of vaccine antigens or therapeutic proteins in transgenic chloroplasts (leaves) or chromoplasts (fruits/roots) and antibiotic-free selection systems available in plastid transformation systems became successful in the oral delivery of vaccine antigens against cholera, tetanus, anthrax, plague, and canine parvovirus [17, 28, 69, 84]. Although higher level protein production is vital of chloroplast, too much expression of foreign proteins in chloroplasts is causing toxicity on host plant. Temporary immersion bioreactors (TIBs) using Alka Burst technology has produced leafy biomass that expressed *OspA* at levels of up to 7.6% total soluble protein to give a maximum yield of *OspA* (about 108 mg/L). These results show that TIBs offer an alternative method for the production of transplastomic biomass proteins, which are non-toxic for plants and particularly useful when absolute gene dispersion control is required [85] From a single plant *Chlanydomonas reinhadtii* various recombinant therapeutic proteins have been produced (Table 5).

**Table 5:** Recombinant therapeutic proteins produced in the chloroplast of *Chlanydomonas reinhadtii*

Therapeutic protein	Expression	References
αCD22HCH23PE40; dimeric version of αCD22PE40	0.2–0.3% TSP	[86]
Human glutamic acid decarboxylase (hGAD65)	0.25–0.3% TSP	[87]
*Escherichia coli* phytase gene (appA)	Not detected	[88]
CtxB-Pfs25; *Plasmodium falciparum* surface protein 25 fused to the β subunit of the choleratoxin from Vibrio cholera	0.09% TSP	[89]
Mammary associated serum amyloid (M-SSA)	3–5% TSP	[90]
αCD22CH23Gel; dimeric version of αCD22Gel	0.1–0.2% TSP	[91]
Infectious burial disease virus (IBDV-VP2)	0.8–4% TCP	[92]

### Phytoremediation

It is strongly believed that phytoremediation is a safe and cost-effective system for cleaning up contaminated environments using plants. Organomercurial compounds are the most toxic forms of mercury and chloroplast genome is a primary target of mercury damage in plants. It is, thus, an ideal site to engineer resistance and detoxification of organomercurials and metallic mercury [93]. Chloroplast genetic engineering of plants for synthesis of metal chelators has improved the capability of plants for metal uptake [94, 95].

Two bacterial genes encoding two enzymes, mercuric ion reductase (*merA*) and organomercurial lyase (*merB*), were expressed as an operon in transgenic tobacco chloroplasts. This demonstrated accumulate of mercury in roots to levels surpassing the concentration in soil, up to 200 μg/g, without any detrimental effect and could accumulate 100-fold more mercury in leaves than untransformed plants [96]. Phytoremediation of toxic mercury was achieved by engineering of tobacco chloroplast with metallothionein enzyme [53].

### Production of industrial enzymes and biomaterials

Chloroplast genome has been successfully engineered to produce important enzymes and biomaterials. Despite the diversion of major metabolic intermediate, metabolic engineering using chloroplast genomes produced the highest level of the poly (*p*-hydroxybenzoic acid (pHBA) polymer (25% dry weight) in normal healthy plants [97]. Optimized genetic construct for plastid transformation of tobacco (*Nicotiana tobacum*) for the production of the renewable biodegradable plastic poly hydroxy butyrate (PHB) was designed using an operon extension strategy [98]. Lots of efforts have been made to produce PHB in different systems, but to date, the highest levels of PHB have been achieved in plastids. This was due to the high flux of the PHB pathway substrate acetyl-CoA through this organelle during fatty acid biosynthesis [99, 100] Typical examples of biomaterials and enzymes that have been engineered via chloroplast genome of Tobacco are mentioned in (Table 6).

**Table 6:** Biomaterials and enzymes engineered via chloroplast genome of Tobacco

Enzymes/biomaterials	Gene	Yield	References
β-glucosidase	*Bgl1*	20 mg g^−1^ TSP	[101]
Elastin-derived polymer	*Eg121*	Not detected	[102]
Fibronectin extra domain A	*EDA*	2% TCP	[68]
Xylanase	*xynA*	6% TSP	[101]
	*Xyn*	35% TSP	[103]
Endo-glucanase	*celB*	60–70% TSP	[103]
Superoxide dismutase	*Cu/ZnSOD*	9% TSP	[97]
Polyhydroxybutyrate	*phb operon*	18.8% TSP	[104]
*p*-Hydroxybenzoic acid	*ubiC*	13–18% TSP	[97]
Cellulases	*bgl1C*, *cel6B*, *cel9A*, *xeg74*	5–40% TSP	[105]
	*CelA*, *CelB*	22–23 mg g^−1^ TSP,	[106]

### Production of biofuels

The most important and first requirement for lingo-cellulosic biofuels production is to develop an efficient enzyme production system for economical and rapid biomass depolymerization. High levels of expression and compartmentalization of toxic proteins within chloroplasts enables to protect transgenic plants from pleiotropic effects, making chloroplast an ideal bioreactor for industrial enzyme production [25]. Although it was possible to have single biofuels enzymes expressed whole biomass hydrolysis was not effective because of the requirement of more number of enzymes [94, 95]. The development of chloroplast derived cocktails of enzymes for production of fermentable sugars from different ligno-cellulosic biomass become major fresh breakthrough in biofuels research. Different enzymes from bacteria or fungi, namely *β*-*1,4*-*endoglucanase*, *Beta* glucosidase, Swollenin, *esterase*, *cutinase*, *endoglucanases*, *exoglucanase*, *pectate lyases*, *xylanase*, *lipase*, *acetyl*, *Acetyl xylan esterase* and *xylan* were expressed in tobacco chloroplasts for production of fermentable sugars [107–111].

*Endoglucanase Ce19A*, *β*-*glucosidase Bg11C*, *Exoglucanase Ce16B* and *xyloglucanase Xeg74* from *Trichoderma fusca* were highly active and hydrolyzed their synthetic test substrates in a dose dependent manner. The cocktail of these enzymes triggered efficient sugar release from straw [107]. Treatment of cotton fiber with chloroplast derived cutinase showed enlarged segments and the intertwined inner fibers were irreversibly unwound due to expansion activity of cutinase. Chloroplast derived cutinase showed esterase and lipase activity [110]. *Β*-*1,4*-*endoglucanase* from *Pyrococcus horikoshii* which drives EPGh from chloroplast was able to recover from dry leaves and digested carboxylmethyl cellulose(CMC) substrate [56]. β-Mannanase enzyme from *Trichoderma reesei* showed sixfold to sevenfold higher enzyme activity than *E. coli*. β-Mannanase enzyme cocktail with chloroplast derived mannanse yielded 20% more glucose equivalents from pinewood than the cocktail without mannanase [111]. Catalytic activity of chloroplast produced *Xylanase* was detected with birch wood xylan as substrate [112]. Chloroplast enzymes (*Endoglucanase*, *Swollenin*, *Acetyl xylan esterase*, *Xylanase* enzymes originated from *T. reesei*, *Endoglucanase exoglucanase* from *C. thermocellum*, *Lipase* from *M. tuberclosis*, *Cutinase* and *Pectate lyase A* from *F. solani*) showed wider pH and higher temperature stability than enzymes expressed in *E. coli.* Chloroplast derived crude extract enzyme cocktails yielded more than 36-fold glucose from citrus peel, filter paper or pine wood than commercial cocktails [113].

## Conclusion and prospects

Chloroplast genome has become the target of many plant genetic transformation efforts due to its enormous advantages over nuclear genome of the plant. The nuclear transgenic approach is incapable to develop products when higher-level transgene expression and multigene engineering is a requirement. Chloroplast transformation is expected to offer unique advantages in the advancement of different biotechnological applications; including, phytoremediation, production of industrial enzymes, biofuels, biomaterials, molecular farming for the production of antibiotics, vaccines, biopharmaceuticals and conferring agronomic traits. Chloroplast transformation has been achieved only to tobacco, lettuce, Arabidopsis, tomato, carrot, oilseed rape, potato, cabbage, cotton, petunia, soybean, sugarcane, sugar beet, rice, eggplant, cauliflower and poplar [114].

Although successful progresses have been made, full potential exploitation of chloroplast technology requires addressing critical challenges. These include: recalcitrant nature of cereal species to existing regeneration protocols is daunting so developing efficient shoot regeneration system is very critical [115], optimizing the level of expression as massive expression of foreign proteins is resulting in phenotypic alterations of transplastomic plants [116], lack of appropriate tissue specific regulatory sequences [117, 118], problem of gene expression in non-green plastids [119], unintended homologus recombination that hinder efficient recovery of transplastomic transformants containing the desired transgene [120], degradation of foreign proteins is a limiting factor for accumulation of foreign proteins in transgenic chloroplasts [50, 121, 122] low frequency transgene dispersion might occur due to occasionally parental/biparental transmission of plastids and via transgene transfer to nuclear genome [115]. To ease public concern and increase public acceptance production of marker free transplastomic plants is also very important. As chloroplast genome is capable of expressing more than 120 foreign genes originated from different organisms (bacteria, animals, viruses, fungi and humans), addressing the above barriers will make chloroplast genome very attractive site for various biotechnological applications with incredible impact on human life.

## Abbreviations

UTR: un-translated region
TLP: total leaf protein
TSP: total soluble protein
TCP: total cell protein
